# Estradiol prevented intestinal ischemia and reperfusion-induced changes in intestinal permeability and motility in male rats

**DOI:** 10.6061/clinics/2021/e2683

**Published:** 2021-04-16

**Authors:** Fernanda Yamamoto Ricardo-da-Silva, Evelyn Thaís Fantozzi, Sara Rodrigues-Garbin, Helori Vanni Domingos, Ricardo Martins Oliveira-Filho, Bernardo Boris Vargaftig, Yanira Riffo-Vasquez, Ana Cristina Breithaupt-Faloppa, Wothan Tavares-de-Lima

**Affiliations:** I Laboratorio de Cirurgia Cardiovascular e Fisiopatologia da Circulacao (LIM-11), Instituto do Coracao (InCor), Hospital das Clinicas HCFMUSP, Faculdade de Medicina, Universidade de Sao Paulo, Sao Paulo, SP, BR.; II Departamento de Farmacologia, Instituto de Ciencias Biomedicas, Universidade de Sao Paulo, Sao Paulo, SP, BR.; III Sackler Institute of Pulmonary Pharmacology, Institute of Pharmaceutical Science, King's College London, London, UK.

**Keywords:** Intestine, Ischemia-Reperfusion Injury, Gastrointestinal Motility, Estradiol, Inflammation

## Abstract

**OBJECTIVES:**

Ischemia and reperfusion (I/R) in the intestine could lead to severe endothelial injury, compromising intestinal motility. Reportedly, estradiol can control local and systemic inflammation induced by I/R injury. Thus, we investigated the effects of estradiol treatment on local repercussions in an intestinal I/R model.

**METHODS:**

Rats were subjected to ischemia via the occlusion of the superior mesenteric artery (45 min) followed by reperfusion (2h). Thirty minutes after ischemia induction (E30), 17β-estradiol (E2) was administered as a single dose (280 μg/kg, intravenous). Sham-operated animals were used as controls.

**RESULTS:**

I/R injury decreased intestinal motility and increased intestinal permeability, accompanied by reduced mesenteric endothelial nitric oxide synthase (eNOS) and endothelin (ET) protein expression. Additionally, the levels of serum injury markers and inflammatory mediators were elevated. Estradiol treatment improved intestinal motility, reduced intestinal permeability, and increased eNOS and ET expression. Levels of injury markers and inflammatory mediators were also reduced following estradiol treatment.

**CONCLUSION:**

Collectively, our findings indicate that estradiol treatment can modulate the deleterious intestinal effects of I/R injury. Thus, estradiol mediates the improvement in gut barrier functions and prevents intestinal dysfunction, which may reduce the systemic inflammatory response.

## INTRODUCTION

Acute mesenteric ischemia is characterized by the interruption of blood flow to the intestine and is most commonly attributed to the obstruction of the superior mesenteric artery (SMA) (1). This difficult-to-diagnose emergency can result in severe local and systemic inflammation and should be quickly treated; moreover, it presents a high morbidity and mortality rate (2,3). Therefore, treatments that can promptly control the progress of inflammatory processes and subsequent tissue injury induced by ischemia and reperfusion (I/R) should be investigated.

Ischemia is characterized by a period of hypoxia, associated with a loss of cell barrier function and an increase in vascular permeability (4). It can result in the disruption of the intestinal mucosal barrier, thus affecting both endothelial and epithelial barriers. During the reperfusion period, inflammation is further exacerbated via the introduction of oxygen, thus leading to the production of several oxygen reactive species while further attracting neutrophils (5). Along with tissue damage, intestinal I/R injury can result in abnormalities in intestinal motility (6).

Studies have revealed that female sex hormones, specifically estradiol, can afford protective effects in the intestines not only in intestinal I/R injury models but also under conditions of trauma-hemorrhage shock. Furthermore, estradiol plays a role in controlling inflammatory markers during intestinal I/R injury (7-9). Indeed, we have previously identified that estradiol treatment attenuated intestinal I/R-induced gut injury in male rats, modulating local chemokine release and leukocyte mobilization (9). However, the mechanism underlying the modulatory role of estradiol with functional aspects of the gut after intestinal I/R injury remains unknown. Thus, the objective of the present study was to further investigate the effects of estradiol treatment on intestinal integrity and function after intestinal I/R injury.

## MATERIALS AND METHODS

### Animals

Adult male Wistar rats (60 days, n=24) were procured from our departmental animal facilities and housed five per cage in a temperature-controlled room (12-h light-dark cycle, 21±2°C), with free access to water and food. This study was approved by the Animal Care Committee of the Institute of Biomedical Sciences, University of Sao Paulo, following the guidelines of the National Council of Animal Experimentation, which regulates animal research according to the Brazilian Federal Law (Report n^o^. 111/10/03).

### Study groups

The animals were randomized into three groups: 1) sham, rats undergoing surgical procedures, with no I/R induction; 2) I/R, rats undergoing intestinal I/R injury; 3) E30, rats undergoing intestinal I/R injury and treated with 17β-estradiol (280 μg/kg, intravenously [i.v.]) 30 min after ischemia induction.

### Intestinal I/R injury rat model

In brief, the rats were anesthetized with ketamine/xylazine (100 mg/kg and 20 mg/kg, respectively, intraperitoneally [i.p.]), and a midline laparotomy was performed to expose the abdominal cavity. Then, the superior mesenteric artery (SMA) was isolated and clamped for 45 min. Intestinal reperfusion was performed for 2h after vascular clamp retrieval (9). Sham-operated rats served as controls. All animals were euthanized with an anesthesia overdose and exsanguinated via the abdominal aorta. No animals were lost due to the I/R model.

### Estradiol treatment and quantification of hormonal serum concentration

Thirty minutes after clamping the SMA (ischemia period), a single intravenous injection of 17β-estradiol (280 µg/kg; Sigma-Aldrich^®^, USA) dissolved in a sterile saline solution (0.9%) was administered. Serum estradiol levels were determined using an EIA kit (Cayman Chemical Company, USA) in samples (n=5) obtained at the end of the intestinal reperfusion period, according to the manufacturer’s specifications.

### Gastrointestinal transit evaluation

Based on the gastrointestinal transit method described by Manara et al. (10), overnight-fasted rats were fed 1 mL of 20% activated charcoal in phosphate-buffered saline (PBS) by oral gavage (Lafan Química Fina Ltda, Brazil). After the I/R injury period, the small intestine was retrieved to measure the total length (from the pyloric sphincter to the ileocecal junction) and the distance traveled by the activated charcoal was assessed (from the pyloric sphincter to the furthest point where 1 cm of continuous activated charcoal moved). Data were recorded as a percentage of the total length (percentage of gastrointestinal transit).

### Intestinal mucosal permeability assay

After initiating the reperfusion period under anesthesia, rats were administered intraduodenal dextran-FITC (50 mg/kg, 4 kD, Sigma-Aldrich^®^, USA) diluted in PBS (11). Following intestinal reperfusion for 2h, blood samples were collected from the abdominal aorta, and serum aliquots were processed and stored in a freezer (-80°C). Dextran-FITC fluorescence was determined spectrophotometrically (495 nm Synergy HTX, BioTek^®^, USA), and values were plotted in a standard curve (2,000-0.24 ng/mL), expressed in ng/mL.

### Expression of endothelin and endothelial nitric oxide synthase in mesenteric vessels

In brief, mesentery samples, frozen immediately after retrieval, were sectioned (8 μm) (Leica CM1850 Cryostat; Leica Biosystems, USA) and fixed in cold acetone (10 min). Endogenous peroxidase blockade was performed, and sections were incubated for 15 min in H_2_O_2_ (2%), followed by non-specific blocking with Tris-Buffered Saline-Tween20 (TBST) containing bovine serum albumin (1%) for 15 min. Next, the sections were incubated for 1 h at 37°C with primary antibodies, either endothelial nitric oxide synthase (eNOS) or endothelin-1(ET-1) (1:100; Abcam, UK). After rinsing with TBST, sections were incubated for 2h at 37°C with horseradish peroxidase (HRP)-conjugated secondary antibodies (1:200; Millipore, USA). HRP-substrate (AEC, Vector Laboratories, USA) was used for staining and hematoxylin for counterstaining. AEC-stained structures were identified using an image analyzer (NIS-elements^®^, Nikon, Japan) and expressed as stained area/total area. Sections incubated in the absence of the primary antibody were used as negative controls for the reaction.

### Serum concentration of inflammatory and tissue injury biomarkers

Serum levels of vascular endothelial growth factor (VEGF), interferon-gamma inducible-protein-10 (IP-10), tumor necrosis factor (TNF)-α, interleukin (IL)-10, IL-1β, and IL-6 were quantified. Analyses were performed using a Milliplex^®^ MAP kit (EMD Millipore Corporation, USA) and expressed as pg/mL. Quantification was performed using the Luminex 200-Software xPonent/Analyst 4.2 version (EMD Millipore Corporation, USA). Lactate dehydrogenase (LDH) and alkaline phosphatase activity were measured as indirect indicators of intestinal I/R injury using commercial kits according to the manufacturer's instructions (Quibasa Química Básica Ltda., Brazil).

### Statistical analysis

All data are presented as the mean±standard error of the mean (SEM) and were analyzed by ANOVA, followed by Tukey-Kramer multiple comparison test or Bonferroni post hoc test. Data analyses were performed using GraphPad Prism version 8.3.1 (GraphPad Software, Inc., La Jolla, CA, USA). Statistical significance was set at *p*<0.05 and is described in the figure legends, whereas the ANOVA *p*-values are shown in the figures.

## RESULTS

### Serum estradiol concentration in male rats

First, we quantified the serum estradiol levels in the study groups. Intestinal I/R injury did not alter the estradiol serum levels, but estradiol-treated rats presented high serum estradiol concentrations (Sham: 55.7±25.17 pg/mL; I/R: 94.81±14.46; E30:17,904±3,652*; *p<0.0001 compared with I/R).

### Effect of estradiol treatment on intestinal transit

**Figure 1:**
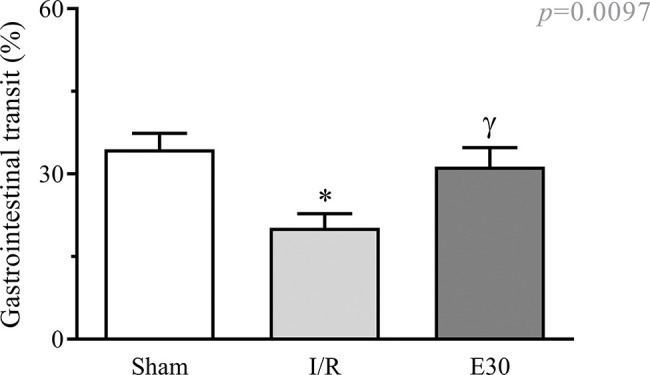
Effect of intestinal I/R injury on the gastrointestinal transit (%). In rats with I/R injury, the superior mesenteric artery was clamped (45 min), followed by intestinal reperfusion (2h). Estradiol (180 µg/kg, i.v.) was administered 30 min after the induction of intestinal ischemia (E30). Sham-operated rats were used as controls. Data are expressed as the mean±standard error of the mean (SEM) from 6 animals. *p=0.011 compared with the sham group; ^γ^*p*=0.0457 *versus* intestinal I/R group. I/R, ischemia and reperfusion.

As shown in Figure 1, intestinal I/R injury significantly reduced gastrointestinal transit when compared with that in animals from the sham group. Estradiol treatment effectively prevented transit reduction, with values similar to those in sham animals.

### Role of estradiol in intestinal mucosa permeability

**Figure 2:**
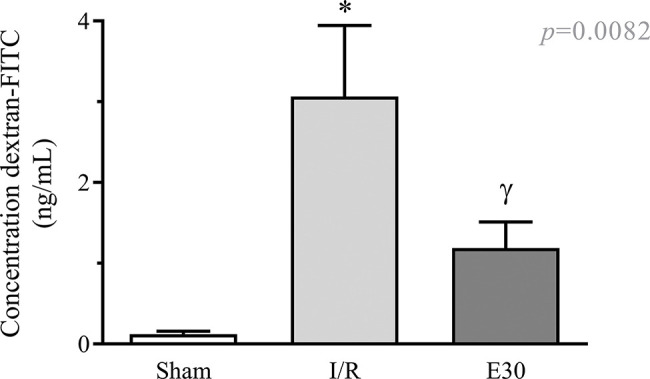
The effect of reperfusion in intestinal I/R on intestinal mucosal permeability. In rats with I/R injury, the superior mesenteric artery was clamped (45 min), followed by intestinal reperfusion (2h). Estradiol (180 µg/kg, i.v.) was administered 30 min after the induction of intestinal ischemia (E30). Sham-operated rats were used as controls. Data are expressed as the mean±standard error of the mean (SEM) from 6 animals. *p<0.0001 compared with sham; ^γ^*p*=0.0036 *versus* intestinal I/R. I/R, ischemia and reperfusion.

As shown in Figure 2, intestinal I/R injury enhanced the permeability of the intestinal mucosa, as observed by the elevated concentration of dextran-FITC in the serum, compared with the sham group. Conversely, estradiol treatment significantly reduced the concentration of serum dextran-FITC compared with that in the I/R group.

### Estradiol treatment and eNOS and ET-1 expression in mesenteric vessels

**Figure 3:**
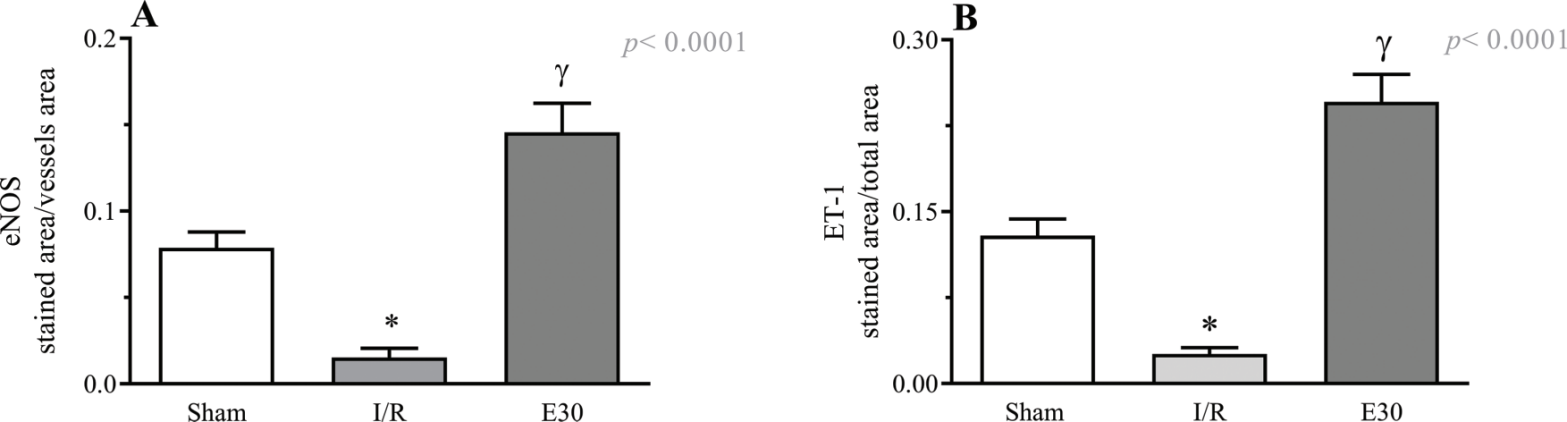
Quantification of eNOS (panel A) and endothelin-1 (panel B) expression in mesenteric vessels. In rats with I/R injury, the superior mesenteric artery was clamped (45 min), followed by intestinal reperfusion (2h). Estradiol (180 µg/kg, i.v.) was administered 30 min after induction of intestinal ischemia (E30). Sham-operated rats were used as control. Data are expressed as the mean±standard error of the mean (SEM) from 4 animals/group (2 tissue sections/animal). Panel A: *p=0.0026 compared with the sham group; ^γ^*p*<0.0001 compared to intestinal I/R. Panel B: *p<0.0001 *versus* sham group; ^γ^*p*<0.0001 *versus* intestinal I/R. eNOS, endothelial nitric oxide synthase; I/R, ischemia and reperfusion.

**Figure 4:**
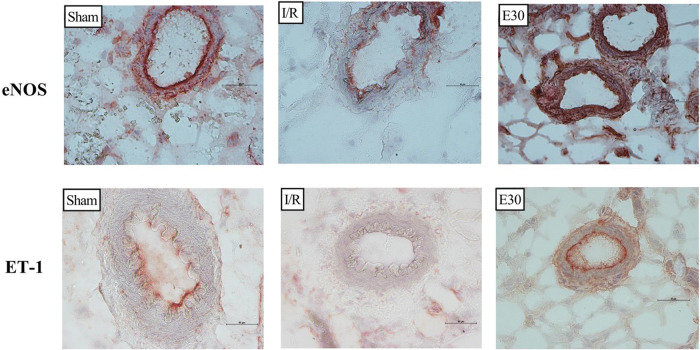
Immunohistochemistry analysis of the expression of endothelial nitric oxide synthase (eNOS) and endothelin (ET-1) in mesenteric vessels. Original magnification, ×40 for all images. In rats with I/R injury, the superior mesenteric artery was clamped (45 min), followed by intestinal reperfusion (2h). Estradiol (180 µg/kg, i.v.) was administered 30 min after the induction of intestinal ischemia (E30). Sham-operated rats were used as controls. I/R, ischemia and reperfusion.

Figure 3 and Figure 4 present the eNOS and ET-1 protein expression levels, respectively, in the mesenteric vessels. Intestinal I/R injury reduced the expression of both eNOS and ET-1, and estradiol treatment (group E30) increased the protein expression levels of both eNOS and ET-1.

### Estradiol treatment and serum levels of injury markers

**Figure 5:**
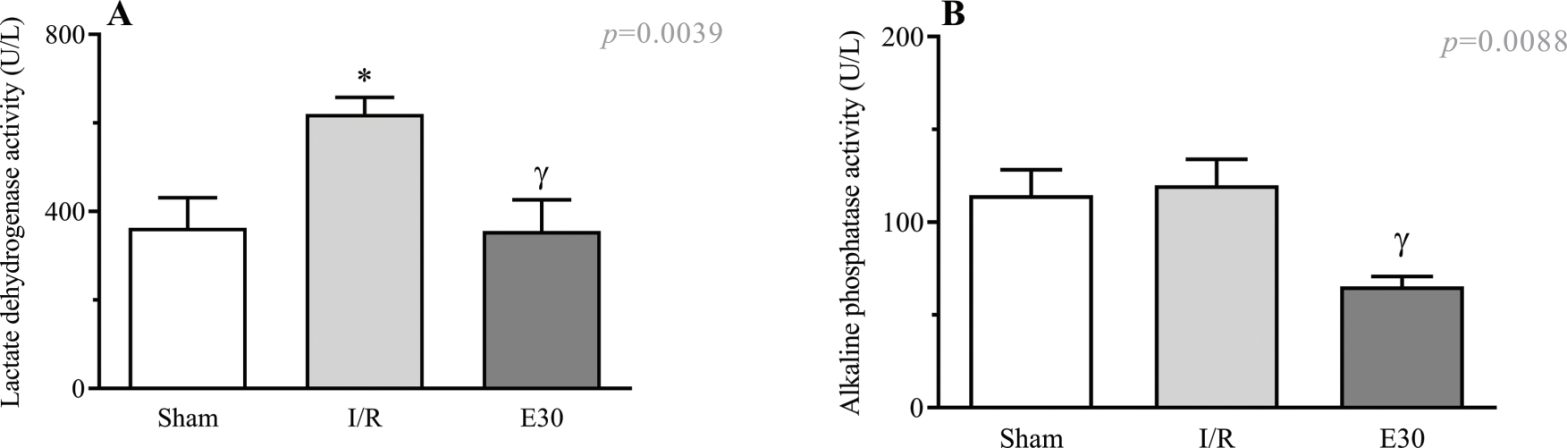
Serum concentrations of lactate dehydrogenase (Panel A) and alkaline phosphatase activity. (Panel B). A group of rats had their superior mesenteric arteries clamped (45 min), followed by intestinal reperfusion (2h). Estradiol (180 µg/kg, i.v.) was administered 30 min after induction of intestinal ischemia (E30). Sham-operated rats were used as control. Data are expressed as the mean±standard error of the mean (SEM) from 8 animals. Panel A: *p=0.0081 compared with sham; ^γ^*p*=0.0066 compared with I/R. Panel B: ^γ^*p*=0.0088 compared with I/R. I/R, ischemia and reperfusion.

Figure 5A shows an increase in the serum LDH levels following intestinal I/R injury compared with the case in the sham group. Estradiol treatment effectively prevented an increase in the LDH levels. Although intestinal I/R injury did not alter the serum levels of alkaline phosphatase, estradiol treatment reduced its concentration (Figure 5B).

### Estradiol-mediated control of the release of inflammatory mediators

**Figure 6:**
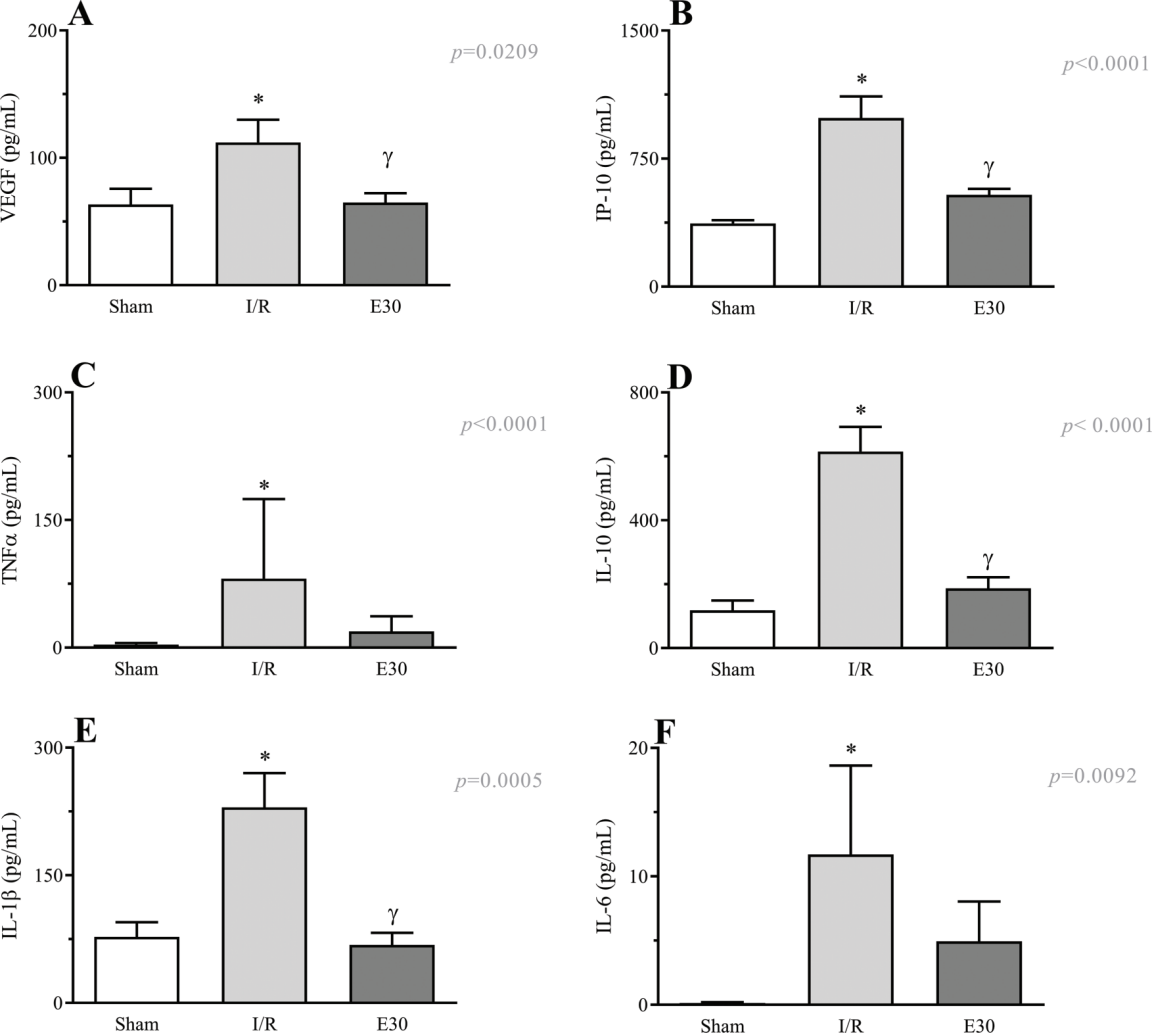
Effects of intestinal I/R injury and estradiol treatment on the serum levels of VEGF (panel A), IP-10 (panel B), TNFα (panel C), IL-10 (panel D), IL-1β (panel D), and IL-6 (panel E). In I/R injury rats, the superior mesenteric artery was clamped (45 min), followed by intestinal reperfusion (2h). Estradiol (180 µg/kg, i.v.) was administered 30 min after induction of intestinal ischemia (E30). Sham-operated rats were used as controls. Data are expressed as the mean±standard error of the mean (SEM) from 8 animals. *p≤0.05 compared with sham; ^γ^*p*≤0.05 compared with I/R. I/R, ischemia and reperfusion; VEGF, vascular endothelial growth factor; IP-10, inducible-protein-10 IL-10, interleukin 10; IL-1β, interleukin-1β; TNFα, tumor necrosis factor-alpha.

As shown in Figure 6 (panels A-F), the induction of intestinal I/R injury increased the serum concentrations of VEGF, IP-10, TNF-α, IL-10, IL-1β, and IL-6. Conversely, estradiol treatment prevented the increase in the expression levels of VEGF, IP-10, IL-10, and IL-1β.

## DISCUSSION

Intestinal I/R accounts for the induction of local, systemic, and remote organ injuries. Toxic products and inflammatory mediators released locally during the ischemic period could reach the systemic compartment during reperfusion, thus impacting the functions of remote organs and becoming life-threatening (12,13). This severe condition appears to be triggered by the intestinal I/R injury, which affects the functionality of intestinal tight junctions (14). The intestinal mucosa acts as a barrier to control the passage of products from the intestine into the systemic compartment (15). Moreover, excessive activation of mucosal permeability by substances translocated from the intestinal lumen has been associated with the induction of gut disease (16).

In the present study, the experimental design addressed the hypothesis that estradiol locally modulates the inflammatory response induced by intestinal I/R injury, and consequently, systemic mediators/markers. Accordingly, we evaluated specific parameters associated with local and systemic inflammatory responses elicited by intestinal I/R injury in male rats. We revealed that tissue injury biomarkers, notably, intestinal permeability and serum levels of LDH, were increased after intestinal I/R injury. Previous studies have reported a loss of intestinal barrier integrity induced by gut ischemic events (17,18), with increased LDH levels detected in the serum after intestinal ischemia (19). In a previous study, we observed that intestinal I/R injury reduces intestinal villous height (9). Therefore, we aimed to investigate the functional aspects of the intestine after I/R injury. Herein, we observed that the intestinal transit distance was reduced in experimental animals. Interestingly, Woting and Blaut (20) have indicated that changes in the intestinal permeability could influence the intestinal transit time. As increased mucosal permeability could account for tissue injury, based on our findings, we speculated whether the reduced intestinal transit could promote a long-lasting interaction between toxic products/inflammatory mediators generated by intestinal I/R injury and intestinal mucosal surfaces, thus contributing to the severity of local inflammation and systemic repercussions. Therefore, estradiol affords protective effects against intestinal transit and could potentially contribute to diminishing this deleterious interaction.

Narita et al. (21) have reported a correlation between the elevated cytokine concentration in the peritoneum after intestinal I/R injury, along with the development of lung inflammation. These authors reinforced the concept of the local influence of intestinal I/R to induce lung inflammation. Using the methodology described by Narita et al. (21), we detected elevated levels of TNF-α and IL-10 in peritoneal fluid collected after induction of intestinal I/R (9), which could explain the gut injury caused by intestinal I/R. Notably, disruption of the gut barrier during intestinal ischemia is associated with the induction of intestinal inflammation, generation of pro-inflammatory mediators, and remote organ failure (22).

Moreover, it is essential to note that blood supply to the mesenteric compartment is finely tuned by local mechanisms (2), and the lack of normal endothelial cell function accounts for the lesion observed after intestinal I/R (4). Thus, we evaluated the functional activity of mesenteric vessels in terms of ET-1 and eNOS expression. Khanna et al. (23) have reported decreased intestinal eNOS activity during intestinal reperfusion. This decrease in NO release can be associated with peroxynitrite production and gut permeability injury (24). In the present study, we detected decreased eNOS expression in the mesenteric vessels, suggesting that intestinal homeostasis was disrupted by mesenteric artery occlusion. Consistently, data from the literature have revealed that intestinal I/R injury increased the plasma levels of ET and modified the mesenteric expression of ET receptors A and B (25). Furthermore, Oktar et al. (26) have shown an increase in intestinal mucosal permeability, neutrophil influx, and free radicals release in the rat mesentery compartment following ET-1 infusion. Our data demonstrated reduced ET expression in mesenteric vessels after intestinal I/R injury. Endothelial cells produce and release ET-1, which, when bound to ET-A receptors, leads to vasoconstriction. Conversely, ET-B receptors located on endothelial cells bind to nitric oxide or increase prostacyclin formation, resulting in vasodilation (27). Moreover, ET has been implicated in the physiological modulation of intestinal motility and secretion (28). Considering these data and the augmented serum concentrations of VEGF, IP-10, IL-1β, and IL-10 after intestinal I/R injury, local control of injury represents an important target to mitigate the remote organ dysfunction observed following intestinal ischemic events.

Several lines of evidence indicate that female sex hormones, notably estradiol, exert an anti-inflammatory role. Indeed, Song et al. (29) have demonstrated that estradiol effectively reduces intestinal injury caused by experimental colitis, decreasing intestinal permeability, nuclear factor kappa B (NF-κB) expression, and increasing mucus generation. Rocha de Sousa et al. (30) have investigated mesenteric injury induced by the obstruction of the aorta in rats. Their findings revealed the preventive action of estradiol whereby it increased the mesenteric eNOS expression, while decreasing the degree of inflammation and mortality. By ameliorating intestinal perfusion, estradiol reportedly improves systemic, cardiac, and intestinal villous injuries observed in an experimental model of trauma-hemorrhage and sepsis (7,31). Moreover, we have previously shown that estradiol protects the intestinal villus against intestinal I/R injury in male rats (9).

Furthermore, estradiol is known to attenuate lung inflammation induced by intestinal ischemia (31-34), and based on our findings, this action could be a consequence of its intestinal effects. As highlighted by Deitch (35), the intestine is the primary target of ischemic events to induce remote organ dysfunction.

In the present study, the protective effects of estradiol treatment were observed even after 30 min of intestinal ischemia and 15 min before intestinal perfusion was re-established. We revealed that estradiol exerted a protective role on the magnitude of the endpoints in relation to intestinal injury, such as LDH and mucosal permeability. Furthermore, estradiol effectively reduced the serum levels of VEGF, IP-10, and IL-1β, indicating a relationship between the protective effects of estradiol on the intestine and the attenuation of the inflammatory responses of remote organs, such as those observed during ischemic events. Furthermore, estradiol restored intestinal transit, possibly improving the functional activity of the intestine and recovering the capacity of mesenteric vessels to express eNOS and endothelin.

From a comprehensive perspective, we inferred that estradiol, by mediating the reestablishment of gut barrier functions and preventing local dysfunction, may reduce the systemic inflammatory response. Therefore, given that remote organ inflammation is a repercussion of local (intestinal) events induced by intestinal I/R injury, our findings hypothesize that the attenuation of remote organ dysfunction may be modulated by the protective effects of estradiol on intestinal lesions.

## AUTHOR CONTRIBUTIONS

Ricardo-da-Silva FY performed the study, analyzed the data, and wrote the manuscript. Fantozzi ET, Rodrigues-Garbin S and Domingos HV contributed to obtaining and analyzing data. Oliveira-Filho RM, Vargaftig BB and Riffo-Vasquez Y contributed to the study concept and manuscript writing. Breithaupt-Faloppa AC and Tavares-de-Lima W designed the study, analyzed the data, and wrote the manuscript.
